# Hypothyroidism impairs the circadian rhythmicity of clock genes and proteins involved in gut nutrient absorption in female mice

**DOI:** 10.3389/fphys.2025.1515437

**Published:** 2025-01-31

**Authors:** Ayla Secio-Silva, Paulo Henrique Evangelista-Silva, Felipe Emrich, Letícia Selvatici-Tolentino, Maíza Ferreira, Ana Bárbara de Paula Silva, Bruno Henrique Gomes, Tatienne Neder Figueira-Costa, André Gustavo Oliveira, Rodrigo Antonio Peliciari-Garcia, Francemilson Goulart-Silva, Paula Bargi-Souza

**Affiliations:** ^1^ Department of Physiology and Biophysics, Institute of Biological Sciences, Federal University of Minas Gerais (UFMG), Belo Horizonte, Brazil; ^2^ Department of Physiology and Biophysics, Institute of Biomedical Sciences, University of São Paulo (USP), São Paulo, Brazil; ^3^ Federal University of Tocantins (UFT), Palmas, Brazil; ^4^ Department of Biological Sciences, Morphophysiology and Pathology Sector, Federal University of São Paulo (UNIFESP), Diadema, Brazil

**Keywords:** thyroid hormones, circadian clock, jejunum, metabolism, female

## Abstract

Hypothyroidism is a common thyroid dysfunction with a higher prevalence in women. Impairments in the regulation of basal metabolism, small intestine nutrient transporter, dyslipidemia, and disruption in circadian clocks have been associated with the thyroid disorder. This study aimed to evaluate whether hypothyroidism affects the small intestine circadian clock and the daily expression pattern of gut nutrient transporters in female mice. Adult female C57BL/6J mice were subjected to hypothyroidism by the administration of methimazole (0.1%) and sodium perchlorate (1%) in drinking water for 45 days. After, the animals were subdivided and euthanized every 4 h over the 24 h period under deep anesthesia. The proximal small intestine segment was collected and immediately frozen for gene expression analysis of circadian core clock components (*Bmal1*, *Per2*, *Cry1,* and *Nr1d1*) and nutrient transporters by RT-qPCR. The daily protein content of nutrient transporters involved in the absorption of the products of hydrolysis of lipids, proteins, and carbohydrates was evaluated over 24 h in isolated small intestinal epithelium by Western blotting. The expression of clock genes and protein content of nutrients transporters in the jejunum of control female mice exhibited a well-defined circadian rhythmicity, while no rhythmic oscillation over 24 h was observed for the transporter transcripts. Hypothyroidism abolished the circadian rhythmicity of circadian clock, punctually reduced the transcript content of *Slc2a5* (GLUT5) at ZT12 and *Slc2a2* (GLUT2) at ZT4, and disrupted the circadian oscillation of L-FABP, CD36, PEPT1, and GLUT2 protein contents in the small intestine of female mice. In conclusion, our findings indicate that thyroid hormones modulate the circadian clock of small intestine and the daily rhythmicity of components related to absorptive processes in female mice. Moreover, our data suggest that the mechanisms triggered by thyroid hormones involve posttranscriptional and/or translational modifications of proteins related to lipid, protein, and carbohydrate absorption. Together, these data contribute to the general comprehension of metabolic alterations often observed in hypothyroidism and have far-reaching implications at clinical levels considering the higher worldwide prevalence of hypothyroidism in women and its association with obesity and metabolic syndrome.

## Graphical Abstract

**Figure:**
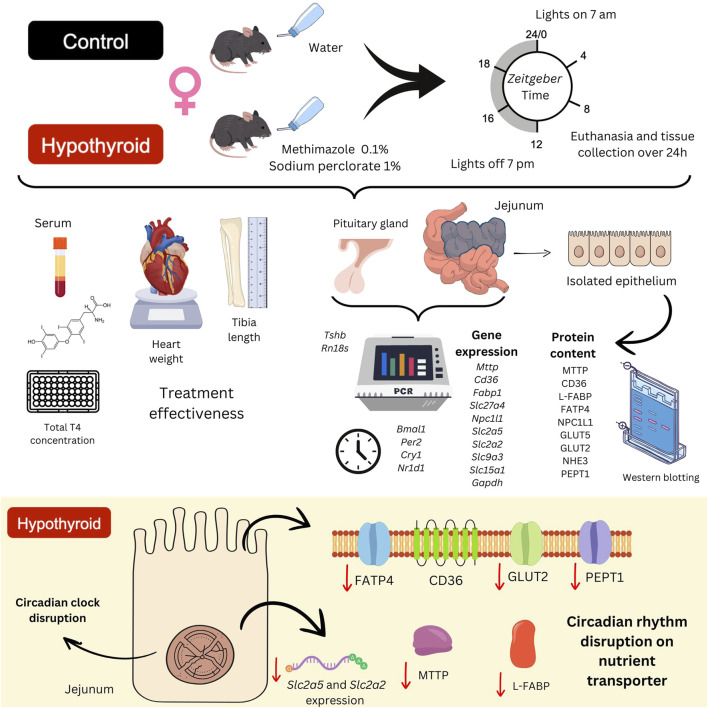
This study aimed to evaluate whether hypothyroidism affects the small intestine circadian clock and the daily expression pattern of gut nutrient transporters in female mice. C57BL/6J adult female mice were divided into Control and Hypothyroid groups. Hypothyroidism was confirmed by the increased *Tshb* mRNA content in the pituitary gland, reduced total thyroxin (T4) serum concentration, and decreased heart weight/tibia length ratio in hypothyroid female mice. The animals were euthanized every 4 h over 24 h under deep anesthesia, and the tissues were freshly collected. Gene expression analysis of the jejunum was performed by RT-qPCR, and the protein content of the isolated intestinal epithelium was evaluated by Western blotting. Hypothyroidism disrupted the circadian oscillation of core clock components and the circadian oscillation of L-FABP, CD36, PEPT1, and GLUT2 protein contents in the small intestine of female mice, which might contribute to the metabolic alterations often observed in hypothyroidism.

## Introduction

The modern lifestyle is associated with exposure to artificial light at night and shift work, leading to the desynchronization of biological rhythms in a significant portion of the population worldwide (Ng and Kaur, 2022). The desynchronization is reinforced by the mealtime irregularity once food exerts an important role as a temporal cue in the entrainment of biological rhythms (Hussain and Pan, 2015; Lewis et al., 2020). The disruption of circadian rhythmicity is strongly associated with impairments in the whole body endocrine-metabolic homeostasis and gastrointestinal processes (Voigt et al., 2019). Higher prevalence of irritable bowel syndrome and abdominal pain are among the major complaints related to gastrointestinal disorders by shift workers and transcontinental travellers (Hussain and Pan, 2015; Knutsson and Boggild, 2010; Nojkov et al., 2010; Vener et al., 1989). Moreover, the disruption of daily rhythmic processes involved in the intestinal macronutrient absorption might contribute to the pathogenesis of endocrine-metabolic disorders (Bishehsari et al., 2016).

The most common endocrine-metabolic diseases are diabetes *mellitus,* metabolic syndrome, and thyroid dysfunctions, showing a positive correlation among them (Khatiwada et al., 2016). Hypothyroidism has a higher prevalence in women, and it is characterized by higher Thyroid Stimulating Hormone (TSH) levels and reduced serum concentrations of thyroid hormones (TH) (Bargi-Souza et al., 2015; Bargi-Souza et al., 2013; Goulart-Silva et al., 2011; Secio-Silva et al., 2022), thyroxine (T4), and triiodothyronine (T3). Moreover, it has been associated with an increased risk of metabolic syndrome development, abdominal obesity, and hypertriglyceridemia in a sex-dependent manner (Iwen et al., 2013; Mehran et al., 2017). Elevated TSH levels, although still within the normal range, are associated with obesity, dyslipidemia, hypertension, inflammation, and metabolic syndrome (Chang et al., 2019). In parallel, individuals with metabolic syndrome exhibit higher free T4 levels and present a positive correlation between insulin resistance and total T3 serum levels (Iwen et al., 2018; Tarcin et al., 2012).

Besides the well-known systemic effects of THs in thermogenesis, energy homeostasis, and metabolism (Iwen et al., 2018; Tarcin et al., 2012; Sentis et al., 2024), THs also affect the intestinal development (Giolito and Plateroti, 2022; Ishizuya-Oka et al., 2009), motility (Xu et al., 2024) and small intestine absorption as described in studies evaluating the jejunum, isolated intestinal epithelium, and enterocytes, as well as in human colon adenocarcinoma cells, known as Caco-2 cells. Briefly, it has been shown that THs increase the villi length (Losacco et al., 2018), modulate the expression of lipid (Losacco et al., 2018; Chu et al., 1995; Shin and Osborne, 2003; Zhu and Cheng, 2010), carbohydrate (Blakemore et al., 1995; Hashimoto et al., 2009; Matosin et al., 1996; Matosin-Matekalo et al., 1999; Mochizuki et al., 2007; Potenza et al., 2009) and protein (Ashida et al., 2002; Ashida et al., 2004; Jumarie and Malo, 1994) transporters by transcriptional and translational mechanisms.

There is a strict correlation between thyroid function and circadian rhythmicity (Ikegami et al., 2019). It has been demonstrated that thyroid axis components exhibit daily rhythmicity (Fliers et al., 2014; Kalsbeek et al., 2005; Kalsbeek et al., 2000; Roelfsema and Veldhuis, 2013). Shift workers have a higher prevalence of hypothyroidism, reaching approximately 30% for women and 20% for men (Oțelea and Călugăreanu, 2018). We have recently demonstrated that thyroid dysfunctions alter the core circadian clock expression in the central clock, located in the suprachiasmatic nuclei (SCN) of the hypothalamus, heart, and pituitary gland (Bargi-Souza et al., 2019; Emrich et al., 2024; Peliciari-Garcia et al., 2018; Peliciari-Garcia et al., 2016). Hypothyroidism also alters the daily pattern of spontaneous locomotor activity, body temperature, and oxygen consumption, showing that THs modulate the rhythmicity of metabolism by mechanisms possibly involving the transcriptional regulation of the core clock component *Bmal1* by T3 (Emrich et al., 2024).

Therefore, considering these THs effects in the molecular clockwork machinery and the increased hypothyroidism prevalence in women, this study aimed to evaluate the rhythmicity of the jejunum clockwork machinery (*Bmal1, Per2, Cry1,* and *Nr1d1*) and transporters involved in the macronutrient absorption (L-FABP, FATP4, MTTP, CD36, NPC1L1, PEPT1, NHE3, GLUT5, and GLUT2) in female mice under control and hypothyroid conditions, looking for a possible explanation for the increased risk of metabolic syndrome development in hypothyroid women, as observed in population studies.

## Materials and methods

### Animals and treatments

Seven-week-old female C57BL/6J mice (*Mus musculus*) were obtained from the Central Animal Facility of the Federal University of Minas Gerais (UFMG). Four to five animals were housed in collective cages (28 cm × 17 cm × 12 cm), with water and food *ad libitum*. They were kept in a temperature (28°C ± 2°C) controlled room under a 12 h/12 h light-dark cycle (07:00 h/19:00 h, Zeitgeber Time (ZT) 0 = 07:00 h). Luminosity was kept between 200 and 300 lux during the light phase and 0.5–1 lux in the dark phase.

The mice were divided into the Control (C - euthyroid) and Hypothyroid (H) groups. Hypothyroidism was induced by the treatment with methimazole (0.1%) and sodium perchlorate (1%), inhibitors of thyroid hormone synthesis, dissolved in the drinking water for 45 days (Emrich et al., 2024; Barros et al., 2023; Marsili et al., 2010). Body weight was weekly evaluated. After treatment, control and hypothyroid animals were equally divided into six subgroups for the 24 h tissue collection, taking place every 4 h, corresponding to ZTs 0/24, 4, 8, 12, 16, and 20, as previously described (Emrich et al., 2024). Each animal was euthanized by decapitation following the inhalation of the anesthetic isoflurane (3%–5%). All experimental procedures were approved by the Ethics Committee on the Use of Animals (CEUA) of the UFMG (CEUA: 349/2023), according to the legislation of the National Council for Control and Animal Experimentation (CONCEA).

### Tissue collection and intestinal epithelium isolation

The pituitary gland was collected for assessment of hypothyroidism induction, immediately frozen in liquid nitrogen, and kept at −80°C for further molecular analysis. The serum was obtained from trunk blood, centrifuged at 4000 RPM for 20 min (5804R Centrifuge, Eppendorf, Hamburg, Germany), and stored at −20°C until subsequent analysis. The heart and tibia were collected for the respective weight and length measurements.

The small intestine segment was collected, and the length was measured from the end of the stomach to the beginning of the cecum. The initial 4 cm corresponding to the duodenum were discarded, and the following 10 cm were collected, which corresponds to the proximal portion of jejunum, which was then washed with saline solution (0.9% NaCl). For the gene expression analysis, 30 mg of tissue was collected, washed, and then frozen in liquid nitrogen and kept at −80°C for further molecular analysis.

For the evaluation of the daily protein content, the jejunum fragment was placed into a washing solution (3.2 mM DTT and 3 mM sodium azide diluted in saline) and processed, as previously described (Losacco et al., 2018; Torelli Hijo et al., 2019). Briefly, the intestine mucosa was exposed, cut into ±0.5 cm pieces, and washed with cold saline to remove the luminal contents. Next, the tissue fragments were incubated in washing solution (0.5 mM DTT and 1.5 mM EDTA, dissolved in saline) for 5 min at room temperature and then bathed in phosphate-buffered saline (PBS, pH 7.4) for 20 min at 4°C. The intestine fragments were incubated in an orbital shaker for 60 min at 37°C and 200 RPM (Orbital shaker, Model 420, ThermoFisher Scientific, United States of America) in a PBS1X solution containing 0.5 mM DTT and 1.5 mM EDTA to detachment of the epithelium. At the end, the liquid phase containing the isolated epithelium was filtered with gauze and transferred to a new tube, followed by centrifugation at 2000 RPM (5804R Centrifuge, Eppendorf, Hamburg, Germany) for 10 min at 4°C. Afterwards, the supernatant was discarded, the pellet was diluted in RIPA lysis buffer (150 mM NaCl, 0.5% sodium deoxycholate, 50 mM Tris·HCl, pH 8.0, 0.1% SDS, 0.1% Nonidet P-40, 2 mM Na_3_VO_4_, 10 mM NaF, and 0.2 mM phenylmethylsulfonyl fluoride (PMSF)), supplemented with complete Mini, EDTA-free Protease Inhibitor Cocktail (Roche Diagnostics), and the homogenate was stored −80°C until protein quantification.

### Total RNA extraction and analysis of gene expression by RT-qPCR

The expression of clock genes and genes encoding proteins involved in nutrient absorption in the jejunum segment, as well as the *Tshb* mRNA content in the pituitary gland, were evaluated using Reverse Transcription (RT) followed by Real-Time Polymerase Chain Reaction (PCR) (RT-qPCR). Total RNA was obtained with TRIzol^®^ reagent (Thermo Fisher Scientific, Waltham, Massachusetts, United States) according to the manufacturer’s protocol. The concentration of total RNA was assessed by spectrophotometry using NanoDrop One (Thermo Fisher Scientific). The integrity of total RNA was also verified from 1 µg of total RNA in agarose gel (1%), stained with GelRed (1:100000, Thermo Fisher Scientific). The total RNA was considered intact when 28S and 18S ribosomal RNA bands were observed in the transilluminator with UV light. The Reverse Transcription assay followed the M-MLV Reverse Transcriptase enzyme protocol (Promega^®^, United States of America) using 1 µg of total RNA of each sample for cDNA synthesis. Real-time PCR was performed using the RT^2^ SYBR^®^ Green qPCR Mastermix (QIAGEN Sciences, Maryland 20874, United States of America) and carried out by ViiA™ 7 Real-Time PCR System (Applied Biosystems™). The primers used for the PCR assay, efficiency, and slope values are listed in Table 1. The specificity of the reaction was analyzed by the dissociation curve (melting point). Efficiency and slope values were determined using a serial dilution curve (0.001 up to 1) (Dussault and Pouliot, 2006). The expression of each target gene in pituitary gland and jejunum was normalized by *Rn18s* and *Gapdh* expression, respectively, according to the 2^−ΔCT^ method (Livak and Schmittgen, 2001; Secio-Silva et al., 2023).

**TABLE 1:** Primer sequences used for qPCR and their respective efficiency and slope values.

Gene	Gene Bank #	Sequence (5′-3′)	Amplicon length (bp)	Slope	Efficiency
*Bmal1*	NM_007489.4	F: GAT GAC GAA CTG AAA CAC R: CTC GGT CAC ATC CTA CGA CAA	70	−3.42	0.96
*Per2*	NM_011066.3	F: GCT GCA GTA GTG AGC AGT CT R: AGG TAA TGC CCT CAA CCT GC	261	−3.27	1.02
*Cry1*	NM_007771.3	F: TTC GCC GGC TCT TCC AA R: ATT GGC ATC AAG ATC CTC AAG A	75	−3.20	1.06
*Nr1d1*	NM_145434.4	F: AGG TGA CCC TGC TTA AGG CTG R: ACT GTC TGG TCC TTC ACG TTG A	82	−3.56	0.91
*Slc9a3*	NM_001081060.3	F: GCA GGA GTA CAA GCA TCT CT R: TCC ATA GGC AGT TTC CCA TTA G	222	−3.01	1.15
*Cd36*	NM_001159558.1	F: GCT AAA TGA GAC TGG GAC CAT R: CAC CAC TCC AAT CCC AAG TAA	118	−3.49	0.93
*Fabp*	NM_017399.5	F: AGG GGG TGT CAG AAA TCG TG R: CCC CCA GGG TGA ACT CAT TG	93	−3.48	0.94
*Slc2a2*	NM_031197.2	F: TCT GTC TGT GTC CAG CTT TG R: CCA ACA TTG CTT TGA TCC TTC C	95	−3.34	0.99
*Npc1l1*	NM_207242.2	F: CCA GAT TAT AGC CTC CCA GTT C R: CCG TAG TTC AGC TGT GAT GT	118	−3.31	1.00
*Slc15a1*	NM_053079.2	F: CAA ACA GTG GGC TGA GTA CA R: GCT GGG TTG ATG TAG GTG TAG	99	−3.62	0.89
*Slc2a5*	NM_019741.3	F: GGT TGG AAT CTG TGC AGG TAT R: GCC GAC AGT GAT GAA GAG TT	118	−3.16	1.07
*Mttp*	NM_001163457.2	F: AGA CCC CTA AGC TCG TTT TCT R: TTT GCT TGG GTT CCT TTCACC	70	−3.49	0.93
*Slc27a4*	NM_011989.5	F: GTG GTG CAC AGC AGG TAT TA R: GTT TCC TGC TGA GTG GTA GAG	111	−3.35	0.99
*Gapdh*	NM_001289726.2	F: GGC AAA TTC AAC GGC ACA GT R: AGA TGG TGA TGG GCT TCC C	70	−3.26	1.03
*Rn18s*	NR_003278.3	F: GCG AAT GGC TCA TTA AAT CAG TTA R: TGG TTT TGA TCT GAT AAA TGC ACG	150	−3.49	0.93
*Tshb*	NM_009432.2	F: GGC AAA CTG TTT CTT CCC AA R: GTT GGT TCT GAC AGC CTC GT	198	−3.13	1.08

### Circadian analysis of protein content in jejunum epithelium

The jejunum epithelium homogenate was centrifuged at 17,949 g at 4°C for 20 min, and the supernatant was collected. Total protein concentration was estimated by spectrophotometry from the bovine serum albumin curve using the Bradford reagent, as described (Bradford, 1976). The samples were then prepared as 1 μg/μL in Laemmli solution. For the SDS-PAGE electrophoresis, 30 µg of each sample were heated to 37°C for 30 min and then applied to SDS-PAGE and subjected to electrophoresis at 100 V for 1 h. In each gel, one sample of each group and respective ZT was applied. Polyacrylamide gels were made at a concentration of 10% for analysis of NPC1L1, NHE3, GLUT2, GLUT5, CD36, MTTP, PEPT1 proteins, and 12% for FATP4 and L-FABP proteins. These intestinal transporters were selected based on their expression and involvement in the absorption of carbohydrates, proteins, and lipids in the jejunum segment. Proteins were transferred by electrophoresis at 100 V for 100 min to a nitrocellulose membrane (Bio-Rad Laboratories, Inc., United States of America). After transference, the membranes were stained with Ponceau S 0.1% (w/v) in 5% (v/v) acetic acid to check equal loading of gels and further data normalization (Romero-Calvo et al., 2010). The labeling specificity for each protein target was confirmed by comparison with the standard molecular weight of Colorcode Prestained Protein Marker (10–180 kDa) (Sinapse Biotecnologia, Brazil) (Table 2), also used as a reference to cut the membranes according to the protein target. The membranes were washed three times with a solution of PBS 1X and 0.1% Tween 20 (PBST) for 10 min each and then blocked with 5% skimmed milk powder diluted in PBST for 1 h at room temperature.

**TABLE 2:** Primary antibodies used for Western blotting.

Protein	Molecular weight (kDa)	Reference	Target Species	Host	Initial concentration	Dilution
L-FABP	14	SC-374537	human, mouse and rat	mouse	200 μg/mL	1:500
FATP4	70	SC-25670	human, mouse and rat	rabbit	200 μg/mL	1:500
MTTP	99	LS-C331668	human, mouse and rat	rabbit	-	1:1000
CD36	88	SC-70644	human, mouse and rat	mouse	200 μg/mL	1:500
NPC1L1	145	SC-166802	human, mouse and rat	mouse	200 μg/mL	1:1000
PEPT1	75	SC-373742	human, mouse and rat	mouse	200 μg/mL	1:500
NHE3	80–100	SC-16103R	human, mouse and rat	rabbit	200 μg/mL	1:500
GLUT5	55	Gtx83627	human, mouse, rat, dog and monkey	mouse	1 mg/mL	1:500
GLUT2	60–62	SC-518022	human, mouse and rat	mouse	200 μg/mL	1:500

The specific primary antibodies used are listed in Table 2. The primary antibody incubation was carried out overnight under agitation at 4°C–8°C. The membranes were then washed four times with PBST, 10 min each, and subjected to incubation with peroxidase-conjugated secondary antibody (Jackson Immuno research laboratories, Inc., United States of America) diluted 1:5000 in PBST for 75 min at room temperature. After this procedure, the membranes were washed four times with PBST and incubated with 1 mL of the Enhanced Chemiluminescence solution (ECL: 1 M Tris-HCL pH 8.5, 250 mM Luminol, 90 mM p-coumaric acid, and 30% hydrogen peroxide). For the GLUT2 and MTTP analyses, the membranes were stripped with 10 mL of stripping solution (Glycine 15 g/mL and NaCl 11.6 g/mL, pH 2.2) for 30 min under agitation, followed by the addition of 625 µL of 1 N NaOH and incubation for another 30 min. Next, the membranes were washed three times with PBST for 10 min. The stripping protocol was repeated twice. After, the membranes were incubated with blocking solution with 5% skimmed milk for 1 h at room temperature, followed by incubation with GLUT2 or MTTP primary antibodies.

The images from immunoblotting membranes were captured in the GE Amersham Image 600 Luminescence Analyzer (GE) instrument (Amersham Biosciences, Amersham, United Kingdom). The densitometry of blots for each target protein per ZT was obtained with ImageJ version 1.53t (Wayne Rasband and contributors, National Institutes of Health, Bethesda, Maryland). Each sample was normalized by the respective lane densitometry of Ponceau staining. The results were expressed as arbitrary units (AU).

### Evaluation of total T4 serum concentration

Total T4 serum concentration was assessed by a commercial ELISA kit (Monobind Inc. - EUA, USA Diagnóstica), according to the manufacturer’s protocol. The absorbance at 450 nm was detected by BioTek Epoch Reader Instruments, Inc.

### Statistical analysis

The outliers were detected by ROUT (Q = 1%) analysis, followed by the normality test using the Shapiro-Wilk test and homoscedasticity using Fisher’s and Spearman’s tests. The comparisons of *Tshb* mRNA content, total T4 serum concentration, and the heart weight/tibia length ratio between Control and Hypothyroid groups were performed using the Mann-Whitney test. Body weight evolution was evaluated by Two-way ANOVA considering the variables treatment (control vs. hypothyroid) and time (weeks), and pairwise comparison was assessed by Bonferroni’s multiple comparison test. The gene expression and protein content data for each group were organized by ZT (0/24, 4, 8, 12, 16, 20). The ZT24 values correspond to the double plotting of the ZT0 results. The oscillation within each experimental group over 24 h was assessed by One-way ANOVA or Kruskal–Wallis tests, according to the criteria of normality and homocedasticity. When One-Way ANOVA or Kurskal-Wallis depicted statistical significance, the time series were subjected to the cosinor fitting of the data with a 24 h periodicity to test whether the depicted oscillation exhibited a circadian pattern (Carvalho-Moreira et al., 2024; Cornelissen, 2014). The rhythmometric parameters (mesor, amplitude, and acrophase) were obtained for the time series that circadian rhythmicity was statistically confirmed. The differences related to the rhythmometric parameters among Control and Hypothyroid groups were compared using the Student’s t-test. Furthermore, Two-way ANOVA was applied considering the variables treatment (control vs. hypothyroid) and time (ZT0/24, 4, 8, 12, 16, and 20), as well as the interaction between these variables. The pairwise comparison was evaluated by Bonferroni’s multiple comparison test. The results were plotted as means ± SEM and considered statistically significant when *P* < 0.05. Statistical analyses were performed using GraphPad Prism version 9.4.1 for macOS, GraphPad Software, San Diego, California, United States of America, www.graphpad.com.

## Results

### Effectiveness of hypothyroidism induction

The effectiveness of hypothyroidism induction was evaluated by the quantification of the thyroid axis components, such as the *Tshb* transcript in the pituitary and total T4 serum concentration. Considering the effects of thyroid hormone on cardiac mass, the systemic hypothyroid condition was assessed by measuring the heart weight (Hajje et al., 2014), which was normalized by the tibia length, once alterations in body weight are expected under hypothyroidism (Rakov et al., 2017; Yin et al., 1982).

**FIGURE 1:**
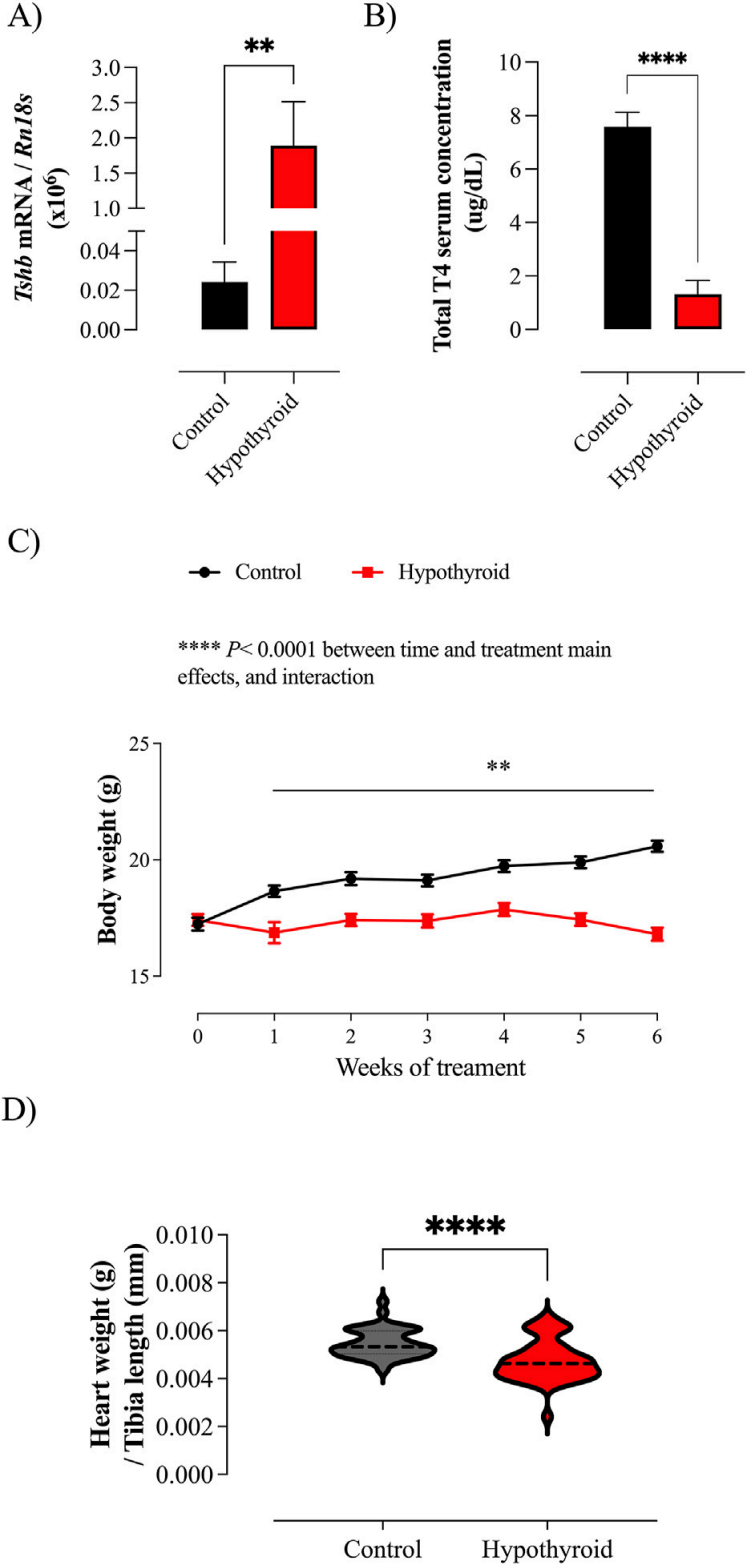
Evaluation of hypothyroidism induction efficacy in female mice and body weight evolution. **(A)** Expression of the *Tshb* transcript in the pituitary gland of Control (black) and Hypothyroid (red) mice. The results were multiplied by 10^6^ for better visualization. **(B)** Total T4 serum concentration in Control and Hypothyroid mice. **(C)** Body weight evolution over the weeks of treatment in Control and Hypothyroid female mice. **(D)** Heart weight/tibia length ratio of Control and Hypothyroid animals. Data are presented as means ± SEM **(A–C)** or median and interquartile range **(D)**. **(A, B, D)** Unpaired *t*-test (two-tailed), **P < 0.01, ****P < 0.0001 vs. Control, *n* = 7–12/group for *Tshb* mRNA expression and total T4 serum concentration; *n* = 43–48/group for heart weight/tibia length ratio. **(C)** Two-way ANOVA considering the variables: treatment (control vs. hypothyroid) and time (weeks of treatment); ****P < 0.0001 for time and treatment main effects (results displayed above the graph). Pairwise comparison was tested by Bonferroni’s multiple comparison test, **P < 0.01 vs. Control group at the respective week of treatment, *n* = 43–48/group.

The hypothyroidism was confirmed by the increased pituitary *Tshb* mRNA content (Figure 1A), reduced total T4 serum concentration (Figure 1B), and heart weight/tibia length ratio (Figure 1D). The body weight of control mice increased over the weeks, while the body weight of the hypothyroid group remained stagnant (Figure 1C).

### Hypothyroidism alters the circadian core clock expression in the proximal intestine of female mice

**FIGURE 2:**
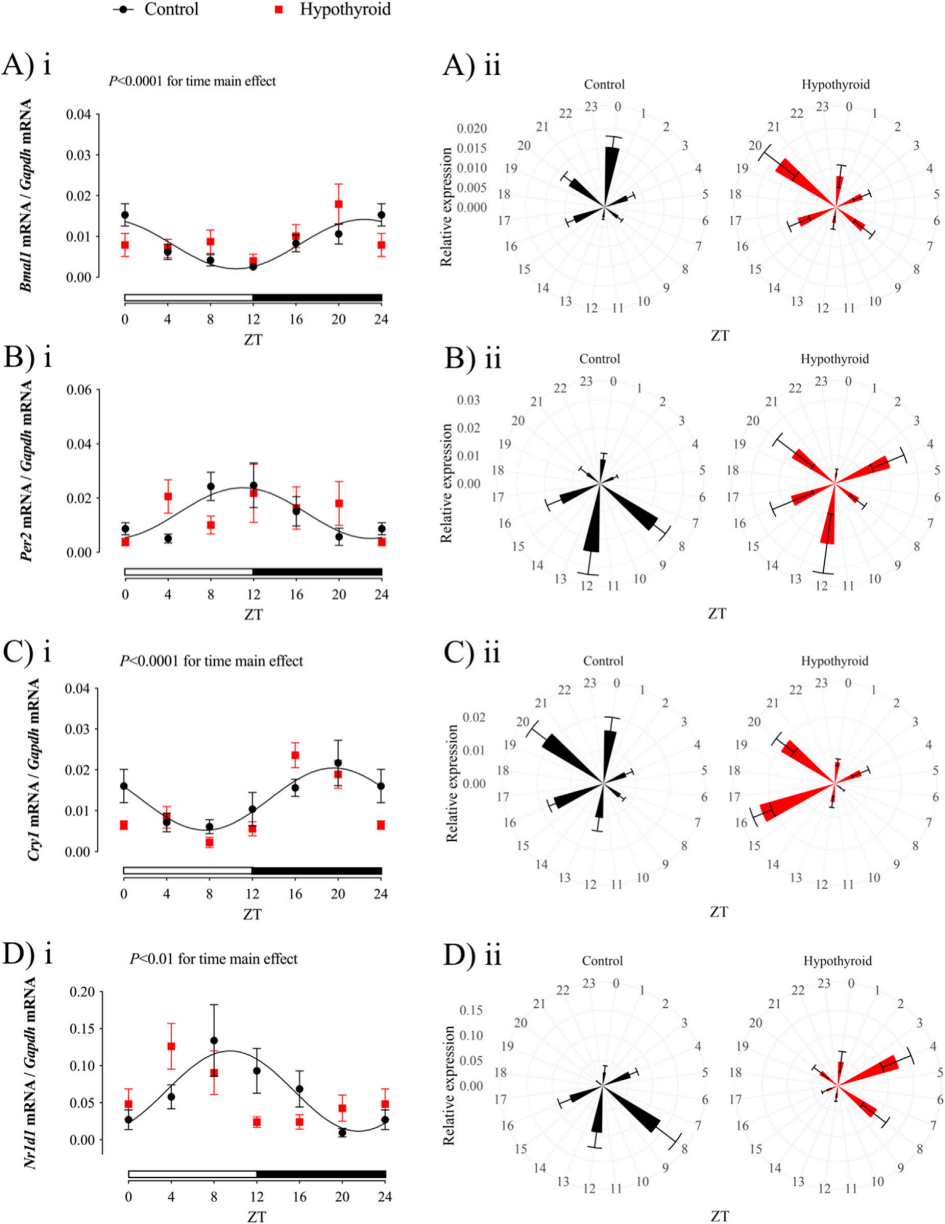
Circadian clock transcript expression in the jejunum of Control (black) and Hypothyroid (red) female mice. Relative gene expression of *Bmal1* **(Ai-ii)**, *Per2* **(Bi-ii)**, *Cry1* **(Ci-ii)**, and *Nr1d1* **(Di-ii)** was normalized by the *Gapdh* mRNA expression. i and ii) Data are presented as means *±* SEM. i) Two-way ANOVA results are described above the graph, and the 24 h cosine curve fitting is represented as black filled lines for the Control group. The absence of a line in the Hypothyroid group indicates no significance for the Kruskal–Wallis test or absence of a 24 h-period of rhythmicity, as described in Table 3. White and black horizontal bars represent the light and dark phases, respectively. ii) Graphical clock representation from the relative expression of each core clock component in the Control and Hypothyroid groups. The ZT24 values correspond to the double plotting of the ZT0 results. *Zeitgeber* Time (ZT); *n* = 6–9/group/ZT.

The expression of core clock components *Bmal1, Per2, Cry1,* and *Nr1d1* was evaluated in the proximal intestine of female mice under control or hypothyroid conditions (Table 3; Figure 2). A significant time variation over 24 h was observed in the expression of all investigated clock genes in the Control group, while only *Cry1* mRNA showed 24 h oscillation in Hypothyroid mice (Table 3, *P* < 0.05 for Kruskal–Wallis test).

**TABLE 3:** Rhythmic analysis of the transcripts’ content in the jejunum of Control and Hypothyroid female mice.

	Kruskal–Wallis test		Cosinor analysis									
	*P*-value		Control					Hypothyroid				
	Control	Hypothyroid	Mesor	Amplitude	Acrophase		*P*-value	Mesor		Amplitude	Acrophase	*P*-value
*Bmal1*	0.0028	0.1989	8.13 ± 0.85	6.05 ± 1.15	22.39 ± 0.79		0.0154	x		x	x	x
*Per2*	0.0231	0.5126	14.51 ± 1.80	9.35 ± 2.43	10.97 ± 1.11		0.0448	x		x	x	x
*Cry1*	0.0454	0.0018	12.84 ± 0.52	7.66 ± 0.75	19.60 ± 0.35		0.0097	-		-	-	0.0974
*Nr1d1*	0.0164	0.0724	65.77 ± 5.99	54.16 ± 8.33	9.512 ± 0.61		0.0072	x		x	x	x
*Fabp*	0.0103	0.6564	-	-	-		0.2598	x		x	x	x
*Slc27a4*	0.0480	0.0413	-	-	-		0.9729	-		-	-	0.2500
*Mttp*	0.3964	0.0670	x	x	x		x	x		x	x	x
*Cd36*	0.6134	0.0683	x	x	x		x	x		x	x	x
*Npc1l1*	0.2630	0.0481	x	x	x		x	-		-	-	0.8430
*Slc15a1*	0.8522	0.7542	x	x	x		x	x		x	x	x
*Slc9a3*	0.9507	0.2208	x	x	x		x	x		x	x	x
*Slc2a5*	0.0839	0.9531	x	x	x		x	x		x	x	x
*Slc2a2*	0.2303	0.1204	x	x	x		x	x		x	x	x

Absence of temporal variation by Kruskal–Wallis test analysis of variance is indicated as “x”. Absence of circadian rhythmicity is represented by “-”. Mesor and amplitude values were multiplied by 1000 for better visualization.

The expression of all investigated core clock components in the proximal intestine of Control female mice exhibited a circadian rhythmicity depicted by the significant *P*-value for the cosine curve fitting of the data (represented by the solid black line in Figure 2; Table 3). No 24 h rhythmicity was detected in the core clock components from the jejunum of Hypothyroid female mice (Figure 2; Table 3).

### Daily expression of transcripts related to nutrient absorption in the jejunum of female mice

**FIGURE 3:**
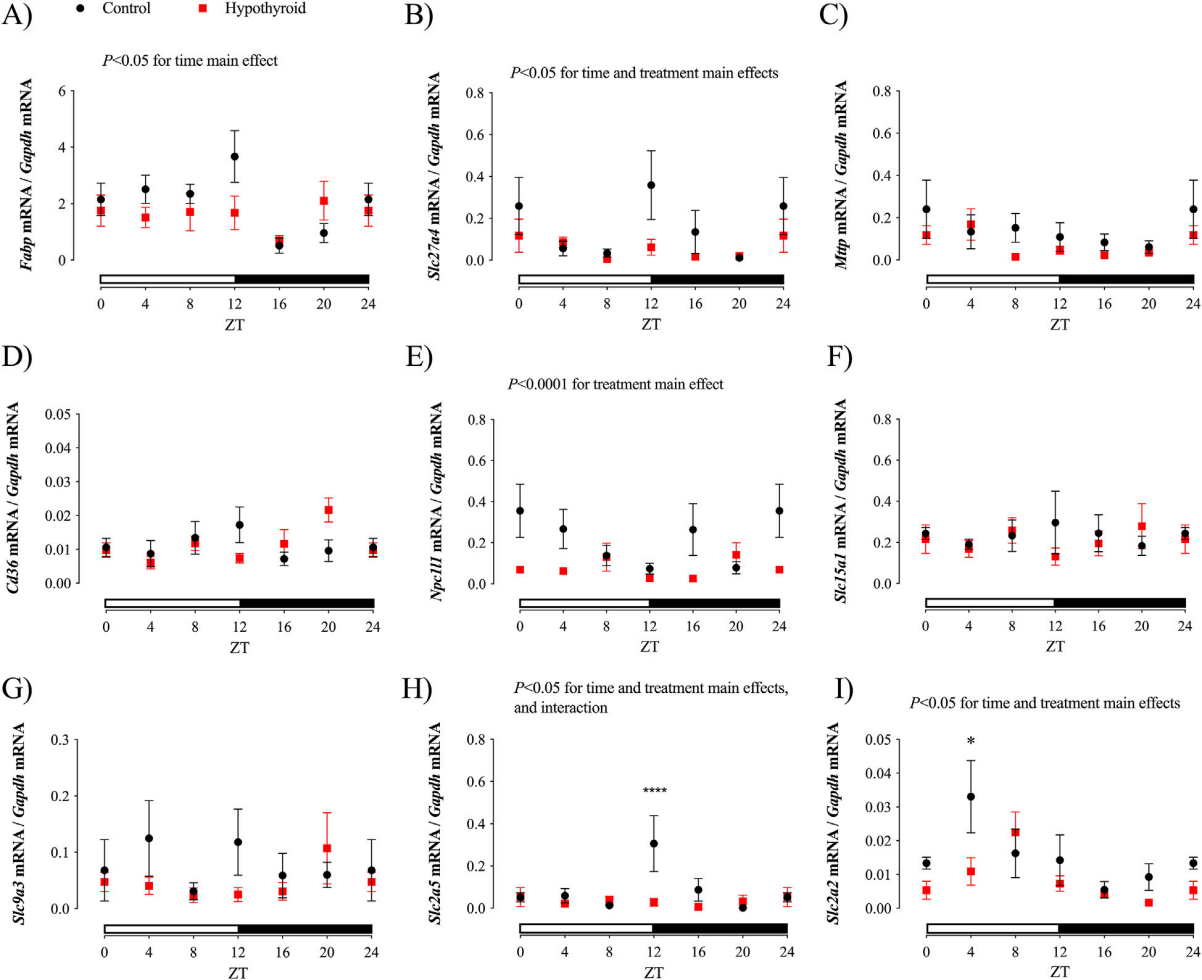
Daily expression of transcripts related to lipid, peptides, sodium, and carbohydrate absorption in the jejunum of Control (black) and Hypothyroid (red) female mice. Daily expression of *Fabp* **(A)**, *Slc27a4* **(B)**, *Mttp* **(C)**, *Cd36* **(D)**, *Npc1l1* **(E)**, *Slc15a1* **(F)**, *Slc9a3* **(G)**, *Slc2a5* **(H)**, and *Slc2a2* **(I)**. The target gene expression was normalized by the *Gapdh* mRNA expression. Data are presented as means ± SEM. Two-way ANOVA results are described above the graph. Bonferroni’s multiple comparison test: *P < 0.05 and ****P < 0.0001 vs. respective Control. The absence of a line indicates no significance for the Kruskal–Wallis test or absence of a 24 h-period of rhythmicity, as described in Table 3. White and black horizontal bars represent the light and dark phases, respectively. The ZT24 values correspond to the double plotting of the ZT0 results. Zeitgeber Time (ZT); *n* = 6–9/group/ZT.

The daily expression pattern of genes encoding proteins involved in the absorption and transport of lipids, peptides, carbohydrates, and sodium in the small intestine of control and hypothyroid female mice was investigated (Figure 3; Table 3).

Significant daily oscillations in jejunum were observed for *Fabp* mRNA content, which encodes FABP, of control female mice; *Slc27a4* gene expression, which encodes FATP4 protein, in both control and hypothyroid mice; and *Npc1l1* mRNA content (encoding NPC1L1) only in jejunum of hypothyroid mice (Table 3). The transcript content of *Fabp, Slc27a4, Slc2a5,* and *Slc2a2,* which encode FABP, FATP4, GLUT5, and GLUT2 proteins, respectively, showed significant time variation in the proximal intestine of female mice (Figures 3A, B, H, I). The effect of treatment (hypothyroidism) was significant for *Slc27a4, Npc1l1, Slc2a2* and *Slc2a5* (Figures 3B, E, H, I). Moreover, the interaction between time and treatment factors was significant for *Slc2a5* (Figure 3H).

No significant alterations were depicted for the content of *Mttp, Cd36, Slc15a1,* and *Slc9a3* transcripts (Figures 3C, D, F, G, respectively), which encode the proteins MTTP, CD36, PEPT1, and NHE3, respectively, according to the Two-way ANOVA. Pair-wise analysis showed punctual differences between control and hypothyroid mice, showing reductions of *Slc2a2* mRNA contents at ZT12 and ZT4, respectively (Figures 3H, I).

According to cosinor analysis, none of these investigated transcripts exhibited a circadian oscillatory pattern in both groups (Table 3).

### Hypothyroidism disrupts the circadian rhythmicity of proteins related to the nutrient transport in isolated intestinal epithelium of female mice

**FIGURE 4:**
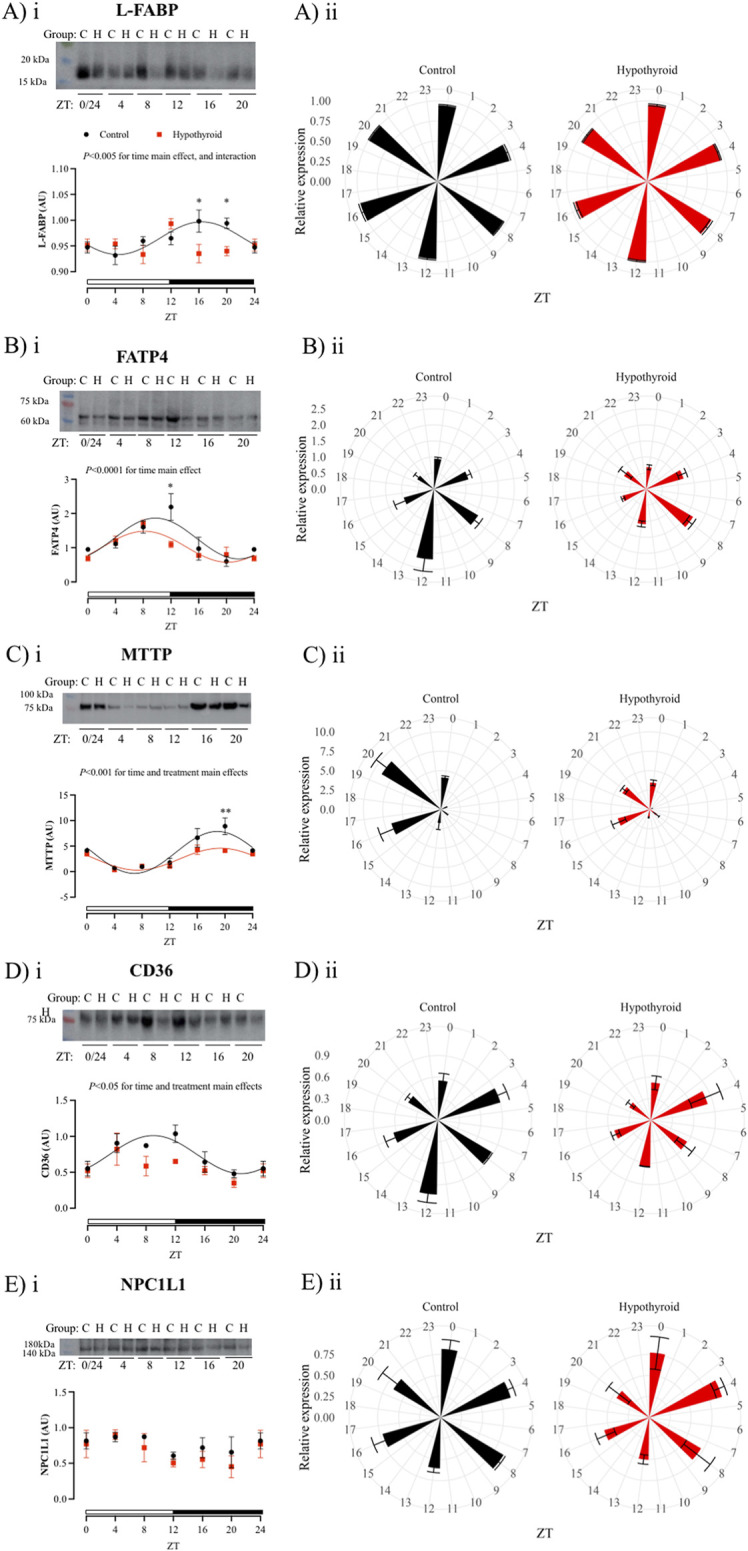
Daily content of proteins related to lipid and cholesterol absorption in the isolated intestinal epithelium of Control (black) and Hypothyroid (red) female mice. Protein content of L-FABP **(Ai-ii)**, FATP4 **(Bi-ii)**, MTTP **(Ci-ii)**, CD36 **(Di-ii)**, and NPC1L1 **(Ei-ii)**, transporters related to the absorption of lipids and cholesterol. i and ii) The data are presented as means ± SEM. i) Representative bands are arranged above the respective graphs, with the molecular weight height indicated alongside. Two-way ANOVA significance is presented above each graph. Bonferroni’s *post hoc* test *P < 0.05 vs. respective control. The 24 h cosine curve fitting is represented as filled lines for the Control (black) and Hypothyroid (red) groups. The absence of a line indicates no significance for the Kruskal–Wallis test/One Way ANOVA or absence of 24 h-period rhythmicity, as described in Table 4. White and black horizontal bars represent the light and dark phases, respectively. ii) Graphical clock representation from the protein content of transporter in Control and Hypothyroid groups. The ZT24 values correspond to the double plotting of the ZT0 results. *Zeitgeber* Time (ZT), *n* = 4–5/group/ZT.

**FIGURE 5:**
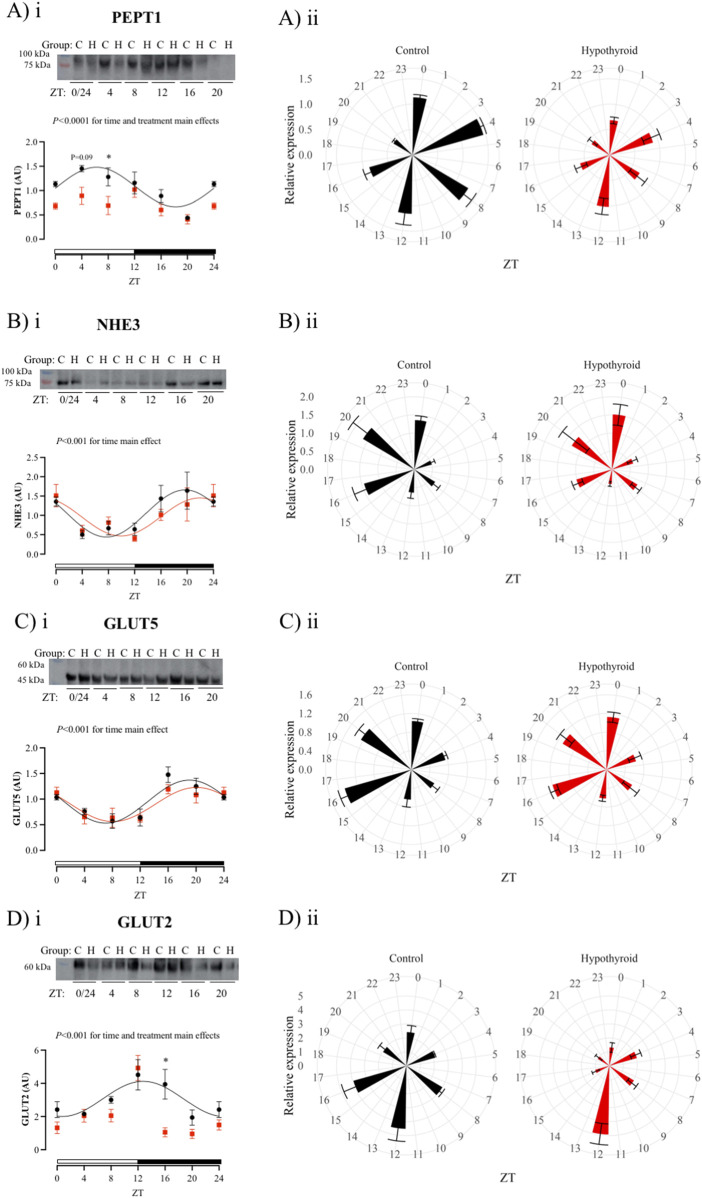
Daily content of proteins related to peptide, sodium, and carbohydrate absorption in the isolated intestinal epithelium of Control (black) and Hypothyroid (red) female mice. Protein content of PEPT1 **(Ai-ii)** and NHE3 **(Bi-ii)**, transporters related to peptide and sodium intestinal absorption, and GLUT5 **(Ci-ii)** and GLUT2 **(Di-ii)** transporters related to the intestinal carbohydrate absorption. i and ii) The data are presented as means ± SEM. i) Representative bands are arranged above the respective graphs, with the molecular weight height indicated alongside. Two-way ANOVA significance is presented above each graph. Bonferroni’s *post hoc* test *P < 0.05 vs. respective control. The 24 h cosine curve fitting is represented as filled lines for the Control (black) and Hypothyroid (red) groups. The absence of a line in the Hypothyroid group indicates no significance for the Kruskal–Wallis test/One-Way ANOVA or absence of 24 h-period rhythmicity, as described in Table 4. White and black horizontal bars represent the light and dark phases, respectively. ii) Graphical clock representation from the protein content of transporter in Control and Hypothyroid groups. The ZT24 values correspond to the double plotting of the ZT0 results. *Zeitgeber* Time (ZT), *n* = 4–5/group/ZT.

The temporal analysis within each experimental group showed significant daily oscillations for all investigated proteins in the isolated intestinal epithelium of female mice, except for CD36 and PEPT1 in the hypothyroid group and NPC1L1 in both experimental groups (Table 4). The content of all investigated proteins, with the exception of NPC1L1 (Figure 4E), exhibited circadian rhythmicity in the intestinal epithelium of the Control group (Figures 4, 5). The respective mesor, amplitude, and acrophase are described in Table 4.

**TABLE 4:** Rhythmic analysis of nutrient transport-related proteins investigated in the isolated intestinal epithelium of Control and Hypothyroid female mice.

	Kruskal–Wallis test or one-way ANOVA		Cosinor analysis								
	*P*-value		Control				Hypothyroid				
	Control	Hypothyroid	Mesor	Amplitude	Acrophase	*P*-value	Mesor		Amplitude	Acrophase	*P*-value
L-FABP	0.0074	0.0204	9.65 ± 0.04	0.32 ± 0.06	16.40 ± 0.71	0.0194	-		-	-	0.8105
FATP4	0.0024	0.0257	12.68 ± 1.23	5.96 ± 1.69	9.753 ± 1.14	0.0496	10.27 ± 0.74		4.54 ± 1.07	8.18 ± 0.86	0.0330
MTTP	0.0166	0.0068	37.62 ± 4.07	41.05 ± 6.08	18.88 ± 0.51	0.0064	24.36 ± 2.78		21.45 ± 4.12*	19.35 ± 0.67	0.0163
CD36	0.0072	0.2516	7.47 ± 0.37	2.647 ± 0.53	8.98 ± 0.76	0.0180	x		x	x	x
NPC1L1	0.4559	0.4519	x	x	x	x	x		x	x	x
PEPT1	0.0019	0.0955	10.74 ± 0.72	4.08 ± 1.08	6.23 ± 0.90	0.0486	x		x	x	x
NHE3	0.0065	0.0362	10.48 ± 0.67	6.10 ± 0.98	19.70 ± 0.57	0.0085	9.59 ± 0.99		4.93 ± 1.36	21.94 ± 1.12	0.0499
GLUT5	0.0140	0.0493	9.52 ± 0.66	4.20 ± 0.98	19.07 ± 0.80	0.0310	8.95 ± 0.51		3.33 ± 0.74	20.09 ± 0.80	0.0260
GLUT2	0.0337	0.0200	30.62 ± 2.22	10.60 ± 2.95	12.76 ± 1.19	0.0482	-		-	-	0.2594

Absence of temporal variation by One-Way ANOVA, or Kruskal–Wallis test analysis of variance is indicated as “x”. Absence of circadian rhythmicity is represented by “-”. Mesor and amplitude values were multiplied by 10 for better visualization. One-tailed, unpaired Student’s t-test (*n* = 4–5/group/ZT) *P < 0.05 vs. respective Control.

Hypothyroidism disrupted the circadian pattern of L-FABP, CD36, PEPT1, and GLUT2 content (Figures 4A, D, 5A, D, respectively; Table 4), and reduced the rhythmic amplitude of MTTP protein (Figure 4Ci; Table 4) in isolated intestinal epithelium of female mice.

The comparison between control and hypothyroid groups showed significance for time main effect in all proteins evaluated (Figures 4, 5), with the exception of NPC1L1. The effects of hypothyroidism (treatment factor) were significant for the content of MTTP, CD36, PEPT1, and GLUT2 proteins (Figures 4C, D, 5A, D), while the interaction between time and treatment was significant only for L-FABP content (Figure 4A). In addition, the hypothyroidism reduced L-FABP content at ZTs 16 and 20 (Figure 4Ai), FATP4 at ZT12 (Figure 4Bi), MTTP at ZT20 (Figure 4Ci), PEPT1 at ZT 8 (while *P* = 0.09 was depicted at ZT4) (Figure 5Ai), and GLUT2 at ZT16 (Figure 5Di) according to the pairwise comparisons.

## Discussion

The present study shows that the proximal intestine of female mice exhibits a functional circadian clock and that the protein content of most nutrient transporters rhythmically oscillates over the 24 h period (except NPC1L1). Moreover, this study demonstrates that hypothyroidism alters the circadian rhythmicity of the jejunum clock and the majority of proteins involved in the absorption/transport of nutrients in the proximal intestine of female mice.

The proximal intestine is an important player in energy homeostasis given its role in macronutrient absorption. This process exhibits daily rhythmicity which in turn, is under control of the endogenous circadian clock but also driven by food intake (Hussain and Pan, 2015; Sladek et al., 2007). Herein, the circadian pattern of core clock gene expression in the proximal intestine confirms the existence of functional clockwork machinery in the jejunum of female mice. Importantly, this transcript expression analysis was performed in the whole jejunum in order to preserve the strict communication between epithelial, immune, and neural cells in the mucosa and muscle and the intrinsic synchronization of the clock genes, which could be compromised by the tissue dissociation and cell isolation (Becquet et al., 2014; de Lima Cavalcanti et al., 2023).

A clear circadian rhythmicity in the protein content of nutrient transporters in control enterocytes was observed, except for NPC1L1. Interestingly, the protein circadian pattern of jejunum nutrient transporters did not correlate with their respective transcript levels. Several studies demonstrate that circadian regulation can occur in different stages of protein expression, from epigenetic modifications to transcriptional, posttranscriptional, translational, and posttranslational regulations (Green et al., 2016; Kojima et al., 2011). For instance, the daily rhythms of one-fifth of proteins in the liver are not accompanied by transcriptional level alterations (Robles et al., 2014). Therefore, the data from control female mice strongly suggest that part of intestine plasticity related to the absorptive processes over 24 h might involve the regulation of steps beyond gene transcription, such as posttranscriptional and/or translation steps. Further studies might help to characterize the molecular mechanisms involved in the circadian regulation of gut transporters.

The enterocytes of the proximal intestine are targets of TH actions (Losacco et al., 2018; Matosin-Matekalo et al., 1999; Mochizuki et al., 2007; Ashida et al., 2002; Ashida et al., 2004; Jumarie and Malo, 1994). The TH are involved in regulation of circadian clockwork machinery in a sex- and tissue-specific manner (Bargi-Souza et al., 2019; Emrich et al., 2024; Peliciari-Garcia et al., 2018; Peliciari-Garcia et al., 2016; Barros et al., 2023; de Assis et al., 2024). It is well known that the desynchronization of biological rhythms affects several physiological and biochemical processes and is associated with a higher prevalence of obesity and metabolic disorders (Antunes et al., 2010). Herein, we have shown that hypothyroidism disrupts the circadian expression of core clock components in the small intestine of female mice. A recent study demonstrated the specific deletion of *Bmal1* in the intestine of mice decreased glucose absorption due to the reduction of SGLT1 protein levels. This effect led to a decline in hepatic glycogen levels and points out the pivotal role of the intestine circadian clock for the systemic metabolic homeostasis (Onuma et al., 2022). Similar correlation could be applied to our data considering the loss of circadian rhythmicity in the expression of core clock components, including *Bmal1*, in the small intestines of female mice.

As observed in control animals, the daily content of all investigated transcripts related to nutrient absorption did not exhibit a circadian pattern in the hypothyroid small intestine. Next, the daily pattern of proteins involved in nutrient absorption was investigated in the isolated epithelium of female mice. It is worth mentioning that the isolation of this epithelium ensures a refinement in the technique ensuring the analysis of transporters exclusively in intestinal sites involved with nutrient absorption. The hypothyroidism disrupts L-FABP, CD36, PEPT1, and GLUT2 circadian rhythms, reduces the MTTP amplitude, and punctually decreases the FATP4 content at the moment of the light to dark photoperiod transition. In parallel, NPC1L1 content in female enterocytes was not altered by hypothyroidism. Together, these findings indicate a possible impairment in the absorption of all macronutrients under hypothyroid conditions.

MTTP, FATP4, CD36, and L-FABP proteins are involved in the intestinal absorption of lipid hydrolysis products (Hussain, 2014). MTTP is a key protein for chylomicron formation, being essential for the processing and absorption of lipid from a diet, and its deficiency in the intestine is associated with lipid malabsorption (Xie et al., 2006; Yen et al., 2015). CD36 protein is an important transporter for the absorption of long-chain fatty acids and regulation of chylomicron formation (Cifarelli and Abumrad, 2018). Also, L-FABP exerts an important role in the lipid absorption, once it binds to lipid metabolites that cross the apical membrane of the enterocyte, transporting them to the endoplasmic reticulum, where they are used for the TAG synthesis, a prerequisite for the chylomicron biogenesis (Gajda and Storch, 2015). Thus, hypothyroidism seems to impact the daily rhythmicity of transport and processing of products of the triacylglycerol hydrolysis, such as fatty acids.

Previous studies have reported that hypothyroid-induced alterations in whole-body metabolism are accompanied by tissue-specific changes in triglycerides-derived fat acid uptake (Klieverik et al., 2009). Downregulation of low-density lipoprotein (LDL) receptor activity, decreased hepatic uptake of cholesterol from the circulation, reduction of T3-mediated control of sterol regulatory element-binding protein 2 (SREBP-2) - which modulates cholesterol biosynthesis through regulation of 3-hydroxy-3-methylglutaryl-coenzyme A reductase (HMG-COA redutase) activity (Duntas and Brenta, 2018) - reduced hepatic β-oxidation and insulin resistance are also associated with hypothyroidism. Together, these alterations might contribute to the increased risk of dyslipidemias and abnormal accumulation of liver triglycerides (hepatic steatosis) often observed in hypothyroid individuals (Hatziagelaki et al., 2022).

Regarding the content of proteins related to peptide absorption, control female enterocytes exhibited a well-defined circadian oscillatory pattern for the PEPT1 and NHE3 proteins, with acrophase in opposition (antiphase). The circadian oscillatory pattern of PEPT1 content and its activity have been previously reported in the jejunum and duodenum portions of the small intestines of rats (Pan et al., 2002; Qandeel et al., 2009). Hypothyroidism does not affect the NHE3 rhythmicity while it abolishes the PEPT1 circadian oscillation. Our findings suggest that the intestinal absorption of dietary protein could be impaired under hypothyroid conditions, which might contribute to the reduction of protein synthesis and muscle weakness observed in such thyroid dysfunction (Mullur et al., 2014; Salvatore et al., 2014).

Finally, regarding the proteins involved in carbohydrate transportation, both GLUT5 and GLUT2 have shown a circadian oscillatory pattern in the enterocytes of control female mice. The acrophase of GLUT2 in the control group occurred at the beginning of the dark phase (ZT12), which is consistent with the activity pattern of nocturnal mice. Similar daily oscillation in the GLUT2 protein was described in the rat small intestine (Corpe and Burant, 1996). Although the intestinal glucose absorption is mainly regulated by the SGLT1 protein, previous studies have demonstrated that in response to increased glucose concentration after a meal, the GLUT2 is translocated to the apical membrane of enterocytes, contributing to the glucose uptake in the small intestine. Besides, GLUT2 present in the enterocyte basolateral membrane of the enterocyte is crucial for the release of glucose into the bloodstream (Kellett and Brot-Laroche, 2005). Our results show that hypothyroidism impairs the daily expression of GLUT2 in enterocytes and may contribute to the carbohydrate-altered metabolism in this thyroid dysfunction.

It is worth mentioning that changes in the proteins related to the absorption of nutrients in enterocytes could influence the plasticity and morphology of the jejunum, as already described in hypothyroid male mice (Losacco et al., 2018), culminating in the shift of preferred metabolic pathways. Together, these alterations could contribute to body composition and metabolic changes under hypothyroid conditions, as commonly observed in malabsorption diseases such as celiac disease (Medza and Szlagatys-Sidorkiewicz, 2023; Montoro-Huguet et al., 2021). It is important to highlight that the identified phenotype was evaluated solely in females, and no functional assays for jejunum nutrient absorption were conducted. Further functional investigations may contribute to the characterization of the small intestine circadian physiology as well as the implications of the chronodisruption induced by hypothyroidism in the pathogenesis of metabolic syndrome in females.

## Conclusion

In conclusion, our findings demonstrate the importance of THs for the proper rhythmic expression of core clock components and proteins related to the absorption and transport of macronutrients as fatty acids, proteins, and carbohydrates, as well as micronutrients as sodium in the jejunum of female mice. Such alterations may contribute to the wide range of metabolic modifications commonly associated with hypothyroidism, explaining, at least in part, the correlation between hypothyroidism, diabetes *mellitus,* and metabolic syndrome.

## Data Availability

The original contributions presented in the study are included in the article/supplementary material, further inquiries can be directed to the corresponding author.
